# Using psycho‐behavioral phenotyping for overweight and obesity: Confirmation of the 6 factor questionnaire

**DOI:** 10.1002/osp4.555

**Published:** 2021-08-27

**Authors:** Robert F. Kushner, Michael M. Hammond

**Affiliations:** ^1^ Department of Medicine and Medical Education Northwestern University Feinberg School of Medicine Chicago Illinois USA; ^2^ Department of Preventive Medicine Northwestern University Feinberg School of Medicine Chicago Illinois USA

**Keywords:** obesity, psycho‐behavioral phenotyping, questionnaire

## Abstract

**Objective:**

Questionnaires that assess dietary habits, eating behaviors, and relevant psychosocial constructs are routinely used in obesity research and clinical practice. The 6 factor questionnaire (6FQ) was previously developed as an assessment tool for psycho‐behavioral phenotyping. The primary purpose of this study was to confirm and validate the original findings in a large diverse adult population.

**Methods:**

A total of 5399 self‐selected participants (mean age of 48 ± 13 years and body mass index of 32 ± 8 kg/m^2^) completed the 6FQ online. The association between self‐reported demographic data and 6FQ responses was assessed using linear regression models.

**Results:**

Mean factor score and odds ratio analyses consistently demonstrated a statistically significant relationship between factors and body weight even after adjusting for age, sex, and race/ethnicity.

**Conclusions:**

Although the study was correlational in design, the results demonstrate that the 6FQ, an instrument that represents multidimensional unhealthful lifestyle patterns associated with diet, physical activity, cognition, and self‐perception worsen with increasing body weight. Psycho‐behavioral phenotyping may be a useful approach when assessing and treating patients with obesity.

## 1 INTRODUCTION

Since obesity is considered a multifactorial disease due to the interaction of various biological, psychosocial, and cognitive factors experienced throughout life,^1^ there is a need to develop a tool that can identify the individuality of these differences for each person and facilitate targeted, evidence‐based weight management strategies. Phenotyping patients based on these characteristics represents a unique method to provide more personalized care. Such a tool could be used by people with overweight or obesity as a guide for selecting a more tailored self‐directed approach to weight loss and by clinicians who can enable the initiation of more effective and efficient weight management counseling.

Multiple questionnaires have been developed and validated to assess psychological, behavioral, or dietary factors used primarily for research in individuals with obesity.^2^, ^3^, ^4^, ^5^, ^6^, ^7^, ^8^, ^9^ However, further development of a comprehensive, short, and clinically useful assessment questionnaire that identifies unique patterns of behavior and cognition would be useful for people with obesity and clinicians alike.

Toward this end, we previously developed and validated by factor analysis a new 27‐item questionnaire using two prospective subject groups (*n* = 640).^10^ In the first validation, the questionnaire was administered to a sample of 298 respondents. Based on the reliability and confirmatory factor analysis after the first validation, a revised 27‐item questionnaire was created that deleted one item and reworded three items. The revised questionnaire was subsequently administered to a second validation sample of 342 respondents. The six distinct factors that were generated had excellent psychometric properties with Cronbach's internal consistency reliability estimates ranging from 0.76 to 0.85 and were statistically associated with higher body mass index (BMI) and BMI classifications. Using a four‐point Likert‐type scale, the self‐administered questionnaire allows individuals to rate their level of agreement to statements that reflect the psychosocial, behavioral, and cognitive issues that may influence body weight management.

The six factors target key principles and strategies that have been shown to be important in weight loss and weight maintenance. The Convenient Diner factor addresses the importance of having a regular meal rhythm, calorie control and awareness, more healthy foods and planning. Awareness and control of convenient dining is another aspect of this factor. The second diet‐related factor, the Easily Enticed Eater, focuses on the importance of food temptation and regulation. Two major themes of this factor are environmental cueing of food intake and the hedonic reward of particular foods. The Exercise Struggler focuses on the importance of physical activity and inactivity in weight management. Individuals who identify with this factor experience various barriers that deter them from increasing physical activity.

The fourth factor, the Fast Pacer, addresses the importance of time management, prioritization in self‐care, and effect of stress. The pace of life in America is increasing which leads some individuals to feel more stressed resulting in unhealthy eating and exercise patterns. The fifth factor, Self‐Critic, focuses on body dissatisfaction and body image disparagement. Body image issues and body dissatisfaction brought about by negative weight stereotypes to themselves can lead to disordered eating patterns, depression, lower self‐esteem, and social withdrawal, often referred to as weight bias internalization.^11^ Lastly, the All‐or‐Nothing Doer is synonymous with dichotomous thinking or lack of moderation. Dichotomous thinking is a form of cognitive rigidity whereby individuals tend to place all experiences in one of two opposite categories instead of a continuum. Individuals tend to view weight loss success and life experiences in general in absolute terms.

As a follow‐up to the original article,^10^ the first author published a popular self‐help book,^12^ and the 6 factor questionnaire (6FQ) was posted online. The availability of the questionnaire to a larger diverse, non‐research group allows the opportunity to confirm and validate the original findings. We hypothesize that the 6FQ scores are directly associated with self‐reported BMI.

## 2 METHODS

### 2.1 Participants, data collection, and measures

The dataset for this study consists of individuals who voluntarily completed the 6FQ online at https://drrobertkushner.com/quiz between October 9, 2019 and September 30, 2020. Awareness and interest in the quiz were prompted by the publication of the book, *Six Factors To Fit: Weight Loss that Works for You!*,^12^ television and radio interviews and other social media outlets. After entering baseline demographic information, participants completed the 27‐item quiz that is scored automatically with results sent back to the individual with a brief interpretation and description of the factors. All respondents agreed to the “Term and Conditions” and “Privacy Policy” information posted on the website that included consent to collection, use of data for confidential research purposes, and opting out of third‐party information sharing. The website uses Wordpress installation, and the quiz uses a plugin called Quiz And Survey Master 7.1.3 to collect answers.

Of the 5578 participants, who were 18 years or older and completed the quiz, 114 were excluded for missing BMI values. Of the remaining 5464 participants, 54 were excluded for having BMI values less than 18.5 kg/m^2^, and 11 excluded for having BMI values greater than 70 kg/m^2^. The remaining 5399 participants were included for analyses. Demographic data collected included self‐reported gender, age, height, weight, race, and ethnicity. Transfer and analysis of the stored dataset was approved by the Northwestern University Institutional Review Board.

### 2.2 Measures

Body mass index was calculated as weight in kilograms divided by square of height in meters. Body mass index was classified into two categories for analysis: healthy weight (between 18.5 and 24.9 kg/m^2^) and overweight or obese (≥25 kg/m^2^). Baseline demographic characteristics were compared between participants in the two BMI categories using generalized linear models for continuous variables and chi‐square tests for categorical variables.

For the continuous variables, the numerical responses to each factor item (0 = don't agree at all, 1 = agree a little, 2 = agree, 3 = strongly agree) were added together to create the factor sum score. The sum scores of each of the six factors were then divided by the number of items assigned to each factor score to create the average factor score. The number of items for the six factors was 4 for Fast Pacer, Exercise Struggler, Self‐Critic, and All‐or‐Nothing Doer; 5 for Convenient Diner; and 6 for Easily Enticed Eater. The average factor scores ranged between 0 and 3. As an example, if the summed score for Easily Enticed Eater was 15, it would be divided by 6 (number of items) yielding an average factor score of 2.5.

For the categorical variables, a percentage factor score was calculated as the summed factor score divided by the maximum factor score, multiplied by 100. Using the example above for the Easily Enticed Eater, 15 divided by a maximum score of 18 yields a categorical score of 83%. A cutoff categorical factor score of >66% was empirically used for positive identification of a factor based on the previous validation study.^10^

### 2.3 Statistical analysis

In these analyses, self‐reported BMI was the outcome of interest and the six‐factor score categories were the exposure of interest. Linear regression models were used to assess the association between each average factor score and self‐reported BMI. Logistic regression models were used to calculate the odds of self‐reported overweight or obesity, by six‐factor score categories, using the healthy weight as the reference group. For both analyses, Model 1 was unadjusted and Model 2 was adjusted for age, sex, and race/ethnicity. Analyses were performed using SAS statistical software (version 9.4, SAS Institute). Statistical significance was defined as *p* < 0.05.

## 3 RESULTS

Participant demographics for the study are displayed in Table 1. Participants were predominantly female (86.9%), Caucasian (80%), with an average age of 48 ± 13 years and a BMI of 32 ± 8 kg/m^2^. Participant distribution by age categories was as follows: <20 (1%), 20–29 (9.2%), 30–39 (18.6%), 40–49 (23.5%), 50–59 (25.6%), and ≥60 (22.2%). Body mass index categories were represented as healthy weight (20.5%) and overweight or obese (79.5%). Participants in the healthy weight group were younger and more likely to be female, compared to participants in the overweight and obese categories. Although African Americans made up 7.4% of the total population, their sample prevalence was lower for the healthy weight group (3.1%) compared to the overweight and obese group (8.5%).

**TABLE 1:** Baseline characteristics of 5399 study participants

Demographic variable	Total	Healthy weight	Overweight or obese	*p*‐value
	*N* = 5399	*N* = 1106 (20.5)	*N* = 4293 (79.5)	
Age (years)	47.9 (13.4)	45.0 (14.5)	48.7 (13.0)	<0.0001
Female (% yes)	4678 (86.9)	1013 (91.8)	3665 (85.7)	<0.0001
Race/ethnicity				
African American	399 (7.4)	34 (3.1)	365 (8.5)	<0.0001
Asian	202 (3.8)	80 (7.3)	122 (2.9)	
Caucasian	4311 (80.0)	911 (82.5)	3400 (79.3)	
Hispanic	310 (5.8)	45 (4.1)	265 (6.2)	
Other	169 (3.1)	34 (3.1)	135 (3.2)	

*Note*: Continuous variables represented as mean (SD) and categorical variables represented as *n* (%).

The relationship between the factor scores and BMI was then assessed by performing two linear regression models (Table 2). The association between average factor scores and BMI was statistically significant (*p* < 0.001) for all six factors even after adjusting for age, sex, and race/ethnicity. In the Exercise Struggler factor, for every unit increase in average score, BMI increased by 3.85 kg/m^2^ after adjusting for age, sex, and race/ethnicity. The associated BMI increases for Convenient Diner and Easily Enticed Eater factors were 3.77 and 3.09 kg/m^2^, respectively.

**TABLE 2:** Association between average factor scores and BMI (kg/m^2^)

Average factor score	Regression coefficient estimate	Standard error	*p*‐value
Model 1			
Convenient diner	3.87	0.15	<0.0001
Fast pacer	1.65	0.13	<0.0001
Easily enticed eater	2.99	0.14	<0.0001
Exercise struggler	3.82	0.13	<0.0001
Self‐critic	2.04	0.13	<0.0001
All‐or‐nothing doer	2.72	0.14	<0.0001
Model 2			
Convenient diner	3.77	0.15	<0.0001
Fast pacer	1.80	0.13	<0.0001
Easily enticed eater	3.09	0.13	<0.0001
Exercise struggler	3.85	0.12	<0.0001
Self‐critic	2.29	0.13	<0.0001
All‐or‐nothing doer	2.64	0.14	<0.0001

*Note*: Average factor score (from 0 = “Don't agree at all” to 3 = “Strongly agree”).

Abbreviation: BMI, body mass index.

Table 3 lists the odds ratios (ORs) and 95% confidence intervals (CIs) for self‐reported overweight or obesity by positive factor score prevalence using the healthy weight BMI category (18.5–24.9 kg/m^2^) as the reference. For each of the six factors, the ORs were statistically significant in both unadjusted and adjusted models (*p* < 0.001). Using this statistical approach, participants within the positive Convenient Diner group had higher risk of self‐reported overweight or obese, compared to participants within the negative Convenient Diner group (OR: 3.47; 95% CI: 2.43, 4.94; *p*‐value: <0.0001), after adjusting for age, sex, race/ethnicity. The ORs were 3.40 and 3.19 for Exercise Struggler and All‐or‐Nothing Doer, respectively.

**TABLE 3:** Odds of self‐report overweight or obese by factor categories

Factor category	Odds ratio (95% CI)	*p*‐value
Model 1		
Convenient diner	3.42 (2.42, 4.84)	<0.0001
Fast pacer	2.07 (1.75, 2.45)	<0.0001
Easily enticed eater	2.66 (2.23, 3.17)	<0.0001
Exercise struggler	3.25 (2.50, 4.24)	<0.0001
Self‐critic	2.11 (1.76, 2.54)	<0.0001
All‐or‐nothing doer	3.05 (2.36, 3.92)	<0.0001
Model 2		
Convenient diner	3.47 (2.43, 4.94)	<0.0001
Fast pacer	2.36 (1.99, 2.81)	<0.0001
Easily enticed eater	2.87 (2.40, 3.44)	<0.0001
Exercise struggler	3.40 (2.60, 4.45)	<0.0001
Self‐critic	2.46 (2.04, 2.97)	<0.0001
All‐or‐nothing doer	3.19 (2.47, 4.13)	<0.0001

*Note*: Model 1: unadjusted; Model 2: adjusted for age, sex, and race/ethnicity.

## 4 DISCUSSION

This study demonstrates significant correlations between the six factors and body weight in a large diverse adult population who voluntarily chose to take the online questionnaire. The results confirm the correlational relationship that was observed in our previously published development and validation study.^10^ We chose to limit our BMI categories into healthy weight and overweight and obese combined in order to avoid misclassifications due to self‐reported height and weight. Although the majority of subjects were female (86.9%) and Caucasian (80.0%), it did include a racially, ethnically, and age diverse population of individuals. This is important since the instrument was designed to be used as an assessment tool for overweight and obesity care. Although the study was correlational in design and causality cannot be proved, the results are consistent with our original hypothesis, that is, the six factors that represent multidimensional unhealthful lifestyle patterns associated with diet, physical activity, cognition, and self‐perception worsen with increasing body weight.

The 6FQ supports two communication approaches intended to facilitate improved health behavior change: patient‐centered care and patient treatment tailoring or segmentation. Patient‐centered care is defined as care provision that is consistent with the values, needs, and desires of patients and is achieved when clinicians involve patients in health‐care discussions and decisions.^13^ Core components of patient‐centered care include communication (sharing information and sensitivity to patient needs), partnership (relationship building), and health promotion (supporting optimal health and care through reflection on the patient's history).^14^ By eliciting the personal habits, attitudes, and emotions of the patient and developing a treatment plan based on these reflections, the 6FQ is intended to create a therapeutic alliance and enable patients to exert control over their health and determinants of obesity.

The second communication approach the 6FQ supports is patient tailoring or segmentation. Segmentation theory tells us that a “one size fits all” approach does not meet the needs of all patients.^15^ Psycho‐behavioral segmentation—or segmenting on the basis of what patients do, think, and believe—would allow the provision of personalized and targeted counseling. From a clinical perspective, segmentation is aligned with the concept of phenotyping, which is the identification of individuals based on a set of characteristics, such as physical features, traits, habits, or attitudes. In obesity medicine, most research has focused on identifying phenotypes based on anthropometric difference such as central or peripheral body fat distribution.^16^ Other research that seeks to identify phenotypes based on personality traits,^17^, ^18^ psychology, or behaviors is less consistent. Nonetheless, there is an ongoing effort to better understand the factors that contribute to individual variability and enable more effective interventions that can be targeted and tailored to the individual.^19^ Two recent reports from the Accumulating Data to Optimally Predict Obesity Treatment project highlight the importance of identifying psychosocial^19^ and behavioral^20^ measures that can be used in adult obesity treatment, in addition to a report from an National Institutes of Health workshop on behavioral and psychological phenotyping of physical activity and sedentary behavior.^21^ It is hypothesized that identification of relevant patient factors would allow the clinician to provide more efficient and effective tailored weight management counseling. The 6FQ was developed to serve this purpose.

Although this study confirmed that a short 27‐item self‐administered questionnaire showed significant correlations between the six factors and body weight in a large heterogeneous population, there are some limitations. All of the data were self‐reported. A systematic review showed trends of underestimating weight and BMI and overestimating height by self‐report.^22^ To overcome this potential error, we simplified our database into only two BMI categories. The majority of the participants were female and Caucasian. Although we were successful in reaching a racially diverse population with varying BMI strata, they may not be representative of all individuals who take the questionnaire. We also do not have data on social determinants of health data that may be related to the factors. As a correlational study, we cannot confirm that the relationship between factors and obesity is causal, although factor scores consistently and significantly increased with increasing body weight. We also did not establish that targeted counseling using the six factors will lead to weight loss. Lastly, we did not demonstrate the utility of the 6FQ in a counseling session.

## 5 CONCLUSION

The study confirms a strong, consistent, and statistically significant association between the six factors and body weight in a diverse self‐selected population. The 27‐item questionnaire identifies six self‐identified factors assessing behavioral, cognitive, and affective factors. It is designed as a self‐help tool or as an intake instrument that may allow clinicians to efficiently and effectively counsel patients on targeted treatment recommendations. Among the multitude of barriers that impact a clinician's decision to engage in obesity care, some of the most important factors are time restraints and lack of clarity on what lifestyle issues to focus on in a particular patient. The 6FQ was developed to directly address these concerns. It is a convenient, short, self‐administered instrument that can be completed prior to the patient encounter; it is quickly scored by the patient or clinician; it targets patients' self‐identified behavioral, cognitive, and affective lifestyle factors related to weight gain; it should allow clinicians to counsel patients on targeted treatment recommendations. Ongoing and future studies will evaluate its utility and effectiveness as a communication and assessment tool in weight management.

## CONFLICT OF INTEREST

The first author (Robert F. Kushner) discloses that the 6 factor questionnaire is included in his book *Six Factors to Fit: Weight Loss that Works for You!* and that he receives royalties from the purchase of the book.
